# Glial Cells and Aging: From the CNS to the Cerebellum

**DOI:** 10.3390/ijms26157553

**Published:** 2025-08-05

**Authors:** Gina La Sala, Donatella Farini

**Affiliations:** 1 Institute of Biochemistry and Cell Biology, Italian National Research Council (CNR), International Campus “A.Buzzati-Traverso”, Via E. Ramarini, 32, 00015 Monterotondo Scalo, Italy; 2 Histology and Embryology Section, Department of Biomedicine and Prevention, University of Rome Tor Vergata, Via Montpellier, 1, 00133 Rome, Italy

**Keywords:** astrocytes heterogeneity, Bergmann glia, CBL, CNS-aging, glial cells, microglia, oligodendroglia, white matter

## Abstract

Among brain regions, the cerebellum (CBL) has traditionally been associated with motor control. However, increasing evidence from connectomics and functional imaging has expanded this view, revealing its involvement in a wide range of cognitive and integrative processes. Despite this emerging relevance, the CBL has received comparatively less attention in aging research, which has focused mainly on other central nervous system (CNS) regions such as the neocortex and hippocampus. This review synthesizes the current evidence on glial cell aging across the CNS, emphasizing how cerebellar circuits follow distinct trajectories in terms of cellular remodeling, transcriptional reprogramming, and structural vulnerability. Recent findings highlight that cerebellar astrocytes and microglia exhibit specific signatures related to aging compared to their cortical counterpart, including moderate reactivity, selective immune response, and spatial reorganization. Cerebellar white matter (WM) undergoes structural alteration, suggesting that oligodendroglial cells may undergo region-specific alterations, particularly within WM tracts, although these aspects remain underexplored. Despite the presence of glial remodeling, the CBL maintains a notable degree of structural and functional integrity during aging. This resilience may be the result of the CBL’s ability to maintain synaptic adaptability and homeostatic balance, supported by its highly organized and compartmentalized architecture. A better understanding of the dynamics of cerebellar glial cells in aging may provide new insight into the mechanisms of brain maintenance and identify potential biomarkers for healthy brain aging.

## 1. Introduction

Aging is a complex and multifactorial biological process that affects all tissues and organs, including the central nervous system (CNS), and leads to progressive changes in structure, cellular composition, and function. Although aging is not a disease per se, it is the main risk factor for neurodegenerative diseases (NDs) and age-associated cognitive and motor decline [1].

Understanding how the brain changes with age has become a major scientific and clinical challenge in light of the continuous increase in global life expectancy. Although neurons have long been considered the main focus of age-related research, recent evidence has shifted attention to the role of glial cells, which are now recognized as highly active and heterogeneous players in brain homeostasis. Astrocytes, oligodendroglia and microglia contribute to essential functions such as synaptic regulation, neurotransmitter recycling, immune surveillance, metabolic support, and maintenance of the blood–brain barrier (BBB) [2].

During aging, these glial cell populations undergo profound transcriptional, metabolic, and morphological remodeling, often preceding or amplifying neuronal dysfunction [3,4,5,6]. Among the CNS regions, the CBL has historically been studied in relation to motor control. However, recent advances in connectome and circuit complexity analysis have challenged the traditional notion of the CBL as a uniform and exclusive motor structure. Instead, it is now considered a highly complex region, characterized by a modular organization, specialized neuron types, dense feedback loops, and non-synaptic signaling mechanisms. Emerging evidence, including the identification of novel interneuron subtypes and ephaptic communication, suggests that the CBL engages in parallel, multilayered signal processing with a level of precision that is comparable to that observed in the cortex [7]. This complexity requires a specialized glial compartment capable of precise support and regulation of different microcircuits.

However, converging data from neuroimaging, injury analysis, and functional connectivity studies indicate the involvement of the CBL in non-motor functions such as working memory, attention, emotion regulation, and language [8,9,10].

Its anatomical connections with the prefrontal cortex, the thalamus, and the basal ganglia suggest an integrative role in sensorimotor and cognitive processes. While most aging studies have traditionally focused on the neocortical and hippocampal circuits, recent advances have pointed to the CBL as susceptible to structural and functional alterations with aging. These aspects deserve specific attention, also because the CBL is altered and possibly involved in age-related NDs.

This review aims to synthesize current knowledge on glial specialization and its alterations during aging, moving from general CNS principles to CBL-specific responses. Evidence from the literature shows that cerebellar glia, particularly astrocytes and microglia, exhibit earlier and more pronounced transcriptional changes related to aging than those of other regions of the CNS [3,4,11,12,13]. These findings suggest that the CBL does not simply mirror CNS aging, but rather follows its own trajectory, shaped by region-specific glia-neuron interactions and metabolic demands. For this reason, elucidating how glial aging contributes to cerebellar vulnerability may provide new insights into the cellular and molecular mechanisms of brain aging and its pathological trajectories and may offer identification of potential therapeutic targets.

## 2. Glial Cells in the CNS

Glia cells represent a heterogeneous and highly specialized population of non-neuronal elements within the CNS, where they perform essential roles in the structural, metabolic, and functional maintenance of neural tissue. Traditionally considered passive support elements, glial cells are now recognized as active and essential regulators of brain function, contributing to synaptic transmission, tissue homeostasis, immune signaling, and cerebrovascular dynamics [14]. They form an integrated and responsive network that ensures stability, plasticity, and adaptability of the CNS under physiological and pathological conditions.

The major classes of glial cells in the CNS include astrocytes, oligodendroglia, microglia, and ependymal cells. These cell populations originate from distinct embryological lineages.

Astrocytes, derived from radial glia cells (RGCs), represent the most abundant glial population and show significant morphological and molecular diversity. These cells play a role in the regulation of ions and neurotransmitter homeostasis, facilitating their recycling and supporting neuronal metabolism. They also contribute to the integrity and function of the BBB. In addition to their homeostatic functions, astrocytes exert fine modulatory control over synaptic activity through Ca^2+^-dependent signaling and the release of neurotransmitters (gliotransmission) [15].

Oligodendroglia, a cell lineage that includes oligodendrocyte precursor cells (OPCs) which mature into oligodendrocytes (OLs), classically responsible for myelination of multiple neuronal axons. This process enables efficient saltatory conduction in neurons and provides metabolic and plasticity support. OPCs persist in adulthood and maintain proliferative and differentiation potential in response to injury or demyelination [16].

Microglia, the resident macrophages of the CNS, derive from myeloid progenitors. They exhibit dynamic surveillance activity and are capable of rapid adaptive phenotypic changes in response to local perturbations. Microglia contribute to synaptic pruning, phagocytosis of apoptotic cells and cell debris, and modulation of inflammatory cascades [17].

Recently, border-associated macrophages (BAMs) have been identified as a new player in CNS immune cells. BAMs are located in leptomeninges and perivascular spaces, where they contribute to the regulation of tissue homeostasis and disease [18]. However, they will not be considered in detail in this review.

Ependymal cells, which line the ventricular system and the central canal of the spinal cord, are ciliated epithelial-like glial elements involved in cerebrospinal fluid (CSF) production and circulation. They contribute to CSF homeostasis by facilitating metabolite clearance and preserving the integrity of the periventricular niche. Although historically less studied, recent evidence suggests that ependymal cells contribute to adult neurogenesis, regulation, and maintenance of ventricular–parenchymal interfaces [19]. Nevertheless, due to the limited evidence on their role in aging, particularly in the CBL, and their non-parenchymal localization, these cells are not addressed further in this review.

### 2.1. Astrocytes

Among the CNS glial cell populations, astrocytes are known for their remarkable adaptability and dynamic properties. Recent advances in molecular profiling and high-resolution imaging have revealed that astrocytes constitute a highly heterogeneous population, encompassing developmental origin, region-specific transcriptional programs, functional specializations and structural adaptations.

Astrocyte identity is established early in development by restriction of the RGC lineage [20].

The ontogenetic patterning is a key determinant of the regional identity of astrocytes shaping their transcriptional programs according to local circuit demands, thus influencing their differential susceptibility to aging and pathology [21,22].

Traditionally, categorized into two major morphological classes: protoplasmic astrocytes, predominant in the gray matter (GM) and fibrous astrocytes (FAs), located in the white matter (WM), these cells differ in structure, distribution, and functional roles [23,24,25]. Protoplasmic astrocytes exhibit highly branched processes and occupy non-overlapping spatial domains in close association with neuronal synapses and blood vessels, playing crucial roles in synaptic modulation, neurotransmitter recycling, and neurovascular coupling [26,27]. Conversely, FAs possess elongated and less branched processes aligned with the axonal tracts and are mainly involved in maintaining the integrity of WM, ion homeostasis, and myelination support [27,28].

They interact with oligodendrocytes, the vasculature, and neurons in Ranvier nodes.

Interestingly, a cluster of astrocytes in the WM of the corpus callosum (CC), but not in the CBL, exhibit high expression of immediate early genes, such as *c-fos* and *c-jun*, related to RGCs, as well as others that are enriched in terms of gene ontology (GO) associated with cell proliferation. In vivo functional experiments confirm the proliferation of astrocytes in WM/CC, but not in cerebellar WM, indicating more complex region heterogeneity of these cells [27].

Beyond the classical dichotomy, recent advances in transcriptomic and spatial profiling studies have revealed a more complex landscape of astrocyte taxonomy. Translating Ribosome Affinity Purification (TRAP) and bulk RNA sequencing (seq) analysis have shown substantial transcriptional differences among astrocyte populations in different CNS regions, including the cortex, the striatum, the thalamus, and the brainstem, correlating with specific local functions [22,29]. Single cell (sc)RNA-seq has further refined this view, identifying distinct subpopulations of astrocytes even within individual regions. For example, in the adult mouse cortex and hippocampus, Batiuk et al. (2020) [30], identified five spatially segregated astrocyte subtypes (AST1–AST5) characterized by distinct gene expression profiles and laminar distribution specialized for local circuit demands such as synaptic function, ionic homeostasis, and immune response. Recent spatial transcriptomic and morphometric analysis in 13 CNS regions, including the CBL, demonstrates that only 20% of the identified genes are common in all regions, thus constituting the core genes for astrocytes [31].

They consist of genes for K^+^ homeostasis, neurotransmitter transport, cholesterol metabolism, and G-protein-coupled receptors. When clustering genes that are differentially expressed in one region compared to the other 12, the CBL results as a distinct cell group.

Interestingly, astrocytes from different regions possess a characteristic morphology associated with a specific molecular signature [31]. The region-specific expression of cytoskeletal and adhesion-related genes, such as *Ezr*, *Fermt2*, *Lama2*, and *Sept4*, has been shown to be associated with astrocyte morphometric parameters (territory size, arbor complexity, and spatial orientation).

Notably, the diversity of astrocytes extends to the subcellular level. Perisynaptic astrocytic processes (PAPs) unsheathe synaptic terminals and exhibit activity-dependent remodeling and locally specialized mRNA translation [32,33]. PAPs differ in transporter expression (*Glt1*, *Aqp4*), cytoskeletal composition, and mitochondrial density [34,35,36], enabling compartmentalized regulation of synaptic activity. These features establish PAPs as key effectors of astrocyte function and potential focal points of vulnerability related to aging.

Recent studies have highlighted that astrocytic identity and transcriptional activity are not determined autonomously by individual cells but instead are dynamically shaped by neuron-derived signals and synaptic activity. Spatial transcriptomic analyses of cortical regions have revealed that neuronal activation triggers the expression of localized astrocyte genes involved in glutamate transport, metabolic support, and ion homeostasis. Synergy between neurons and astrocytes is critical for circuit plasticity and memory consolidation [37] and appears to decline with age [34].

Functionally, astrocyte specialization is reflected in several critical physiological processes.

They modulate neuronal activity and synaptic plasticity through Ca^2+^-dependent signaling mechanisms. Intracellular Ca^2+^ dynamics, mediated by astrocytic channels and receptors such as RYR3, regulate gliotransmission and neuronal synchronization [38].

Astrocytes also support neuronal energetics by supplying glycolysis-derived lactate to active neurons [39,40]. Astrocytes are also crucial regulators of CNS immune responses. Under pathological or traumatic stimuli and during aging, they acquire reactive phenotypes dichotomized into neurotoxic A1, secreting pro-inflammatory cytokines, chemokines and complement components and neuroprotective A2 states, secreting neurotrophic factors [41]. This classification will certainly be overcome by the specific transcriptomic analyses that are beginning to broaden the heterogeneity of the functional states of these cells, as defined by the genes they express in different non-physiological contexts [42,43]. Finally, astrocytes contribute to cerebrovascular homeostasis.

Their perivascular endfeets interact with endothelial cells and blood vessel pericytes to regulate blood flow, BBB permeability, and nutrient delivery, forming a key component of the neurovascular unit (NVU) [38].

### 2.2. Oligodendroglia

Oligodendroglia is a cell lineage primarily enriched in WM, where they sustain lifelong myelin production. The regulated differentiation pathway of this lineage includes OPCs, pre-myelinating oligodendrocytes and OLs [44,45,46]. During differentiation, these cells undergo morphological changes: OPCs are prevalently bipolar, while mature cells become highly branched.

Distinct molecular signatures accompany this morphological heterogeneity at each stage of maturation, as revealed by scRNAseq or single nuclear RNA sequencing (snRNAseq) analysis [47].

OPCs are classically identified by the expression of the *Pdgfrα* and the proteoglycan NG2, while OLs characteristically express myelin-associated proteins such as MBP, MAG and MOG.

OPCs are distributed in both WM, often along the axonal tracts, and GM, near the neural soma.

WM OPCs differentiate into OLs more efficiently than their GM counterparts, many of which remain NG2^+^ progenitors [48], and in these two regions they acquire distinct morphological features and transcriptional profiles [49,50,51].

In the adult brain, OPCs maintain mitotic activity to preserve the pool of progenitors and are essential for myelin maintenance and regeneration, since new OLs are continuously generated by their differentiation [52]. This process occurs in response to neuronal activity associated with learning [53,54] and following myelin injury. In this context, OPCs became activated through interaction with microglia and astrocytes [55], proliferate and migrate to the site of injury where they differentiate into OLs [56]. Conversely, in the adult mouse brain, it has been demonstrated that OPCs are essential to maintain the homeostatic state of microglia [57]. OPCs modulate neural activity and contribute to synapse formation by releasing neuromodulatory factors. They are involved in synaptic pruning and axonal remodeling engulfing these neural portions [58,59]. OPCs also support the integrity of the BBB through interaction with endothelial cells, pericytes and astrocytes within the NVU [60].

Finally, these cells exhibit immunomodulatory properties, responding to brain insults and influencing immune cell activity [61]. The traditional view of OPCs as mere progenitors of OLs is challenged by mounting evidence that highlights their dynamic nature and multifaceted contributions in physiological and pathological contexts [16]. scRNAseq has revealed a transcriptional continuum that spans OPCs, pre-myelinating and OLs reflecting progressive states of differentiation [47,62]. Furthermore, mature OLs can be subdivided into transcriptionally distinct populations with regional heterogeneity in terms of their abundance and response to injury [47,50,62]. Recently, Heo et al. (2025) [47] extended the profiling to the human brain, showing that cycling, committed, differentiating, and quiescent OPCs exhibit conserved gene expression signatures with their murine counterpart. This suggests evolutionary conservation in the molecular programs that guide the maturation of OPCs.

In addition to organizing myelin sheaths around axons, OLs play fundamental roles in modulating their plasticity which is important to adapt neural circuits to experiences and acquisition of new skills underpinning learning and memory. Modifications of myelin throughout the life are very dynamic and regulated and include changes in myelin sheath thickness, distribution, and length of the Ranvier nodes [54,63,64]. Alterations in these processes may contribute to age-related cognitive decline.

OLs, in addition to their traditional function in myelination, also provide metabolic support and regulate neuronal activity through their complex interaction with neurons, other glial cells, and the microenvironment [65,66]. For instance, these cells contribute to the maintenance of extracellular ion homeostasis, especially K^+^, through the activation of the voltage-gated ion channel [67] in collaboration with astrocytes. In turn, astrocytes, which highly express genes that control cholesterol metabolism, support OLs in axonal myelination providing this fundamental myelin component, especially during the stage of remyelination [68,69].

### 2.3. Microglia

Microglia, involved in the immune surveillance of neural tissue, develop during embryogenesis from hematopoietic precursors in the yolk sac instead of the neuroectoderm [70,71]. Thereafter, these cells colonize the CNS before differentiation of the BBB and appearance of the other glial cells.

Microglia are considered long-lived and self-renewing cells that exhibit region-specific turnover rates in the brain in both mice and humans [72,73]. These characteristics result in the accumulation of age-related damage over time and the development of a senescence phenotype in these innate immune cells. In the developing and early postnatal brain, microglia exhibit an amoeboid morphology, are highly mobile and, through phagocytosis of dying neurons and secretion of multiple factors, contribute to modeling the neural architecture. Adult cells acquire a ramified and flexible morphology, which is suitable for dynamically surveying their surroundings and interacting with neighboring cells.

They actively participate in the maintenance of CNS homeostasis and the shaping of neural circuits throughout life [74]. For instance, they regulate synaptic plasticity through different signaling pathways, and alteration of these processes can result in aberrant neural functionality [75].

Furthermore, recent studies with leveraging advanced single-cell technologies have revealed the presence of multiple microglial states in both mouse and human brains associated with development, aging, and disease processes, accompanied by the acquisition of distinct morphologies [76,77,78,79,80,81]. These cells can adapt their states in response to a multitude of cues. Consequently, it is a simplistic approach to consider only the “resting” and “activated” polarization scheme (reviewed in ref. [17,82]). As is also evident in other glial cells, specifically astrocytes and oligodendroglia, microglial states are determined by a complex interplay of intrinsic (species, sex, genetic background, age) and extrinsic factors (spatial location and interactions with other cells, local environment). These factors dynamically influence the cells, making it more difficult to gain a deeper understanding of their roles in health, aging, and disease.

## 3. Cerebellar Glial Cells

The glial cells of the CBL display distinctive functional and morphological characteristics.

The CBL is anatomically divided into ten lobules (I–X) and exhibits a trilaminar cortical organization. This consists of a subpial molecular layer (ML), which is rich in interneurons, glial processes, and a network of neuronal extensions, a Purkinje cell layer (PCL), with a monolayer of GABAergic Purkinje cells (PCs); and a granular layer (GL), which contain densely packed granule cells (GCs).

Neuroanatomical and clinical studies support also a functional division of the CBL into a sensorimotor territory, encompassing the anterior lobe and lobule VIII, and a cognitive territory, including posterior lobules VI–VII, particularly Crus II, which are connected to prefrontal and posterior parietal cortices [8,83].

PCs constitute the sole output of the cerebellar cortex, projecting to the deep cerebellar nuclei (DCN), which are embedded within the WM and are involved in highly organized feedback loops.

These neurons integrate excitatory input from climbing fibers (CFs), which originate in the inferior olive (IO) and parallel fibers (PFs) from GCs which in turn are activated by mossy fibers (MFs) arising from different brain regions.

The output of PCs is finely regulated by local inhibitory interneurons located in the ML, which are themselves activated by collaterals of CFs and PFs. Beyond this laminar structure, the CBL exhibits a highly ordered modular organization, defined by parasagittal longitudinal zones. These modules, molecularly marked by the expression of *Aldolase C* (*Aldoc*, or *Zebrin II*), reveal an alternating *Zebrin*-positive (*Z*^+^) and *Zebrin*-negative (*Z*^−^) bands of PCs, corresponding to distinct functional modules with specific input-output relationships and physiological roles [84].

This modular architecture supports distinct sensorimotor and cognitive tasks and aligns with region-specific vulnerability patterns during aging.

Alongside its modular neuronal architecture, the CBL contains a distinct and regionally specialized glial compartment, including astrocytes, oligodendroglia, and microglia that are key elements in the broad array of cerebellar functions.

### 3.1. Astrocytes

The spatial and functional complexity of the CBL is paralleled by a rich and regionally diverse glial landscape, in which astrocytes and their specialized subtypes play essential roles in synaptic regulation, ionic homeostasis, neurovascular coupling, and local circuit modulation [31,85,86]. Within the cerebellar cortex, astrocytes can be categorized into at least four morpho-functionally distinct populations that reflect the laminar organization of the CBL.

Bergmann glia (BG), located in PCL, possess elongated radial processes that extend through the ML and interact closely with PC dendrites [87]. The term Velate astrocytes (VAs) is used to refer to a specific type of astrocyte that is found in the GL. FAs, which occupy the WM and provide structural and metabolic support to myelinated axons, are also present.

Vascular astrocytes, which are distributed across the cortical and subcortical regions, interface with the microvasculature to support BBB integrity and neurovascular coupling [38,85,88,89]. In addition to their morpho-functional diversity, molecular profiling studies demonstrate that cerebellar astrocytes are transcriptionally distinct from their telencephalic counterparts. This distinction is characterized by a unique signature including genes prevalently expressed by BG, such as the regulator of glutamate transport *Sept4* [31].

Among cerebellar astrocytic populations, BG have been the most extensively characterized in terms of molecular identity, morphology, and function. These cells in PCL form overlapping territories and functional microdomains, performing critical roles throughout life. Both BG and other astrocytes originate from RGC progenitors in the ventricular zone (VZ) [85]. While BG retain their radial morphology and settle in PCL, other astrocytes migrate to the ML or GL. During cerebellar development, BG provide structural scaffolding for the expanding cerebellar plate and support the migration of GCs to the ML [90,91]. Postnatally, they contribute to the growth and remodeling of PC dendrites, as well as the stable formation of synaptic connections [92,93,94]. 

In adulthood, BG remain functionally active by maintaining structural integrity and regulating the synaptic microenvironment [95,96].

Their processes are enriched with AQP4 which is involved in the regulation of cellular volume, and KIR4.1 which is involved in ion buffering [86].

Furthermore, BG express the glutamate transporters GLAST, and GLT-1 as well as Ca^2+^-permeable AMPA receptors (AMPAR) and GABA-A receptors which enables them to respond dynamically to glutamate and GABA during synaptic activity [92,97]. 

BG processes tightly unsheathe PC synapses, thus contributing to ion buffering, neurotransmitter clearance, and glutamine production [95].

Ultrastructural analyses have revealed that BG PAPs alter their position in response to synaptic stimulation. This indicates a dynamic interaction with cerebellar synapses that facilitated the clearance of glutamate spill-out, enabling a higher level of regulation of this neurotransmitter than in other regions of the brain [98]. In vivo Ca^2+^ imaging reveals that BG exhibit localized and wave-like Ca^2+^ transients modulated by PF input, noradrenaline and AMPA receptors.

snRNAseq across all cerebellar lobules revealed a region-specific transcriptional heterogeneity in BG cells. A subpopulation, identified by specific genes as well as *Mybpc1* and *Wif1* [99], is enriched in lobules VI, IX, and X, suggesting that BG may exhibit distinct molecular profiles depending on lobular identity [87]. Such heterogeneity may reflect differential functional roles and vulnerability to aging.

Furthermore, topographic studies show that BG are organized into parasagittal domains aligned with PC compartments which are defined by markers such as *Hsp25* [99,100]. This supports the notion of a shared modular organization between glia and neurons [100]. Additionally, the identity of astrocytes is shaped by the presence and activity of neurons rather than being determined autonomously. Neuronal input regulates the maturation of BG via NOTCH signaling, ultimately affecting neurotransmitter uptake and metabolic functions [101,102]. This pathway is conserved in human development, but declines with age, suggesting a deterioration in neuron-astrocyte cooperation over time. Emerging evidence supports the notion that the coordinated interactions between PCs, BG, parenchymal astrocytes, and oligodendroglia are essential for proper cerebellar maturation and long-term structural resilience. For instance, the secretion of SONIC HEDGEHOG (SHH) by PCs induces the diversification of the molecular profiles of BG and VAs which express the SHH receptors (PTCH1/2) differentially [89].

VAs are morphologically distinct from BG displaying a stellate morphology. Structurally, they do not ensheathe individual synapses, but instead surround GC clusters and wrap around glomeruli (MF terminals, Golgi neuron boutons and granule dendrites), without penetrating them [97,103]. VAs display spontaneous Ca^2+^ activity and Ca^2+^ waves, which are stimulated by purinergic signaling [97], suggesting that they modulate cerebellar processing in a fundamentally distinct manner to BG. However, their exact roles remain to be defined.

Overall, cerebellar astrocytes, and BG in particular, emerge as transcriptionally and functionally specialized glial subtypes that are tightly integrated into modular cerebellar circuits and are actively shaped by neuronal signals. Their unique roles in synaptic and metabolic regulation highlight the need to explore astrocyte diversity and vulnerability in the aging CBL in more detail.

### 3.2. Oligodendroglia

The majority of cerebellar OPCs in mice, determined by *Olig2* expression, originate from precursors in the neuroepithelial region of the caudal midbrain and the ventral rhombomer1 and migrate to the cerebellar primordium [104,105]. In this first wave of cells, new OPCs are added from the cerebellar VZ and the fourth ventricle after birth. In humans, Zhong et al. (2023) [106] use scRNAseq to reconstruct the developmental OPC pathway and identify cells positive for *Olig1* and *Pdgfra* in one of the progenitors clusters isolated from the VZ, the same germinal niche of PCs, astrocytes, and BG.

OLs begin to differentiate in the regions surrounding the DCN, which comprise a mixture of WM and GM. They then move progressively to the periphery in parallel with the formation of the cortical lobes, occupying both the axial WM and the cortical layers [86]. Mature OLs and myelination appeared during the first postnatal days. These newborn cells predominantly myelinate calbindin-positive PC axons in a retrograde manner (from their terminal field to the cell body) [86], and this specificity is maintained in mature WM [107]. The molecular cues and signaling pathways that guide the selective myelination of PCs from OLs during cerebellar development and in the adult remain to be clarified.

Myelination of PC axons is necessary for precise synaptic transmission to DCN neurons. In a rat model in which genetic deletion of MBP is followed by severe myelin deficiency, the loss of PC action potential (AP) synchronization is evident, followed by hyperexcitability of postsynaptic DCN neurons [108]. Furthermore, compact myelin is also fundamental to the health of PC axons and to the synapse maintenance. In the same rat model, fewer functional synapses are observed, in addition to anatomical alterations in the presynaptic terminals and a reduction in the number of active zones [108]. These findings have significant implications for understanding the cellular and synaptic basis of motor deficits in demyelinating diseases such as Multiple Sclerosis (MS), where cerebellar involvement and synaptic pathology have been observed in DCN [109].

Using rabies virus-mediated single oligodendrocyte labeling, and transgenic mouse lines, Battulga et al. (2024) [107] show that the differentiation of OPCs in adult WM and GL is mainly directed toward sheathing the afferent CFs from IO and MFs from different regions of the brain.

PFs from GCs in ML are never myelinated [110], even though OPCs are dispersed in this region and extend numerous processes among PC dendrites. These ML processes receive direct synapses from PFs and CFs [111]. It has been suggested that these innervations may provide an inhibitory signal to prevent further differentiation of OPCs in this region of the cerebellar cortex devoid of myelin [111].

PCs are the only output of the CBL, and their neuronal afferents are uniquely organized, receiving thousands of PFs from GCs and a single CFs from the IO. Uncorrected activity in these circuits can affect other connected brain structures, ultimately affecting motor and cognitive behavior. In this regard, the importance of myelin plasticity in neural circuit homeostasis is becoming evident [64,112]. An example of this adaptive response has been demonstrated in CFs.

These axons provide instructive error signals in various forms of learning and adaptive motor response by coding sensory input and generating synchronized signals in a small number of PCs [113,114]. Different CFs must also ensure synchronization if the length of their axons varies in relation to the distance and folding of different folia. To compensate for this, axons are myelinated to a different extent by OLs with longer internodal lengths, and fewer Ranvier nodes, making the conduction time uniform [115]. This response is crucial for the coordinate movements and could be affected by age alongside the reduced ability of older OPCs to differentiate and myelinate,

As in other CNS regions, OLs play a key role in maintaining neural circuits and long-term neuronal integrity. Furthermore, PCs have an exceptionally high metabolic demand due to the high number of excitatory synaptic connections. OLs support axonal energy metabolism by detecting axonal spiking and increasing glycolytic activity, suppling lactate and pyruvate in synergy with astrocytes [66,116,117]. This OL activity decreases with age, probably contributing to axonal abnormalities and increased vulnerability to excitotoxicity in PCs during cerebellar aging [118].

### 3.3. Microglia

Microglia in mouse and human are characterized by molecular heterogeneity in different CNS regions [119,120]. In the CBL, these cells are distributed across the cerebellar layers and contribute to multiple essential functions (reviewed in [121]). During development, microglia shape cerebellar connectivity by clearing apoptotic PCs [122] and regulating the elimination of CF-PC synapses [123]. Like other glial cell populations, cerebellar microglia exhibit specific characteristics. They are less densely localized than in other CNS regions, presenting decreased cell volume, less ramified morphology, and reduced branching sites. Conversely, cerebellar cells have a higher lysosomal content, often engulfed by nuclear debris, and exhibit high clearance activity efficiently phagocytizing apoptotic cells in vitro [124].

Different high-throughput approaches reveal that cerebellar microglia are characterized by differential gene expression patterns compared to other microglial cell populations residing in different CNS regions, in both mice and humans [11,78,80,119,120,124].

Grabert et al. (2016) [11] define the adult mouse cerebellar microglia as a cell population in a more immune-alert state, characterized by a higher expression of genes involved in pathogen/self-recognition, cell adhesion, chemotaxis, signaling integration, antigen presentation, and microbial killing/sequestration. Additionally, Ayata et al., (2018) [124], using a microglia-specific TRAP approach that ensures non-specific activation of microglia, shows that cerebellar cell population is characterized by a reduction in homeostatic microglia genes, which are normally expressed in a “physiological context”. Instead, genes for endocytosis, phagocytosis, and apoptotic cell clearance are upregulated.

The spatial heterogeneity of adult CNS microglia populations remains a subject of debate, partly due to the artifactual activation caused by the cell isolation technique and the selection of the marker genes used to enrich microglia in various scRNAseq studies. Li et al. (2019) [120] reported that several genes previously attributed to regional microglial heterogeneity [11] were also expressed by non-microglial cells. Nonetheless, cerebellar microglial heterogeneity has also been investigated through other approaches, including electrophysiological measurement and the assessment of morphological and epigenetic features. In vivo imaging studies in mice have demonstrated that cerebellar microglia exhibit distinct morphological and dynamic profiles compared to their cortical counterpart with region-specific phenotypes across cerebellar layers.

Nevertheless, these cells have a markedly less ramified morphology compared to their cortical counterparts, they respond to focal laser-induced injury with speed and directionality comparable to those of cortical microglia [125].

Morphological analyses have also identified differences in microglial density and process motility in the cerebellar cortex [125,126]. While the functional implications of these layer-specific patterns remain unclear, microglia in ML and PCL closely contact PC dendrites and the soma, suggesting an active role in monitoring these key neuronal elements.

The specific identity of cerebellar microglia is likely stated by local developmental cues and persists in adulthood, supporting the plasticity of cerebellar circuits. This regional specificity of the cerebellar microglia is also evident in aging. Importantly, these cells exhibit higher basal turnover rates and are more reactive to pathological stimuli compared to microglia in other regions of the CNS [11,73]. Inflammatory signaling within cerebellar microglia has been shown to enhance PC dendritic excitability, in contrast to the reduction in intrinsic excitability observed in cortical neurons under similar conditions [127]. Aberrant immune responses in PCs are also mediated by the activation of BG [128], leading to altered functional connectivity and behavioral changes, including deficits in social interaction, exploratory activity, and the emergence of repetitive behaviors [127].

## 4. CNS Aging

### 4.1. Hallmarks of CNS Aging

The aging of the CNS is a multifactorial and progressive process, marked by cumulative alterations at the anatomical, functional, cellular, and molecular levels. These alterations affect cognitive performance, synaptic plasticity, and neuroglial homeostasis. Macroscopic signs of CNS aging, such as regional atrophy and impaired connectivity, reflect underlying biological dysfunctions and often precede overt pathology. The most evident sign of atrophy is progressive reductions in both GM and WM volume, cortical thinning, sulci widening, and ventricular enlargement [129].

Although neuronal loss appears limited in many regions of the brain, extensive structural remodeling is observed, including dendritic retraction, spine loss, and synaptic reorganization, particularly affecting long-range projection neurons. These morphological changes are correlated with decreased expression of synaptic proteins and neurotransmitter receptors, ultimately impairing synaptic efficacy and interregional communication [130].

WM is particularly vulnerable to age-related degeneration. Magnetic resonance imaging (MRI) studies consistently report selective alterations in anterior brain regions, including the prefrontal cortex, anterior CC, and thalamic radiation, tracts critical to cognitive processing [131,132]. These tracts are among the last to undergo full myelination during neurodevelopment and among the first to degenerate with aging. Advanced diffusion tensor imaging (DTI) analyses in older individuals reveal a progressive reduction in fractional anisotropy, an established marker of microstructural integrity of WM, particularly affecting major associative pathways [133,134]. This alteration of WM leads to compromised cortico-cortical and cortico-subcortical connectivity which is associated with slower information processing and cognitive decline [135,136].

Functionally, aging is associated with a progressive loss of synchrony across the major brain networks. Resting-state functional MRI (rs-fMRI) studies consistently demonstrate that communication between brain regions becomes less coordinated with age. In parallel, aging is commonly associated with slower processing speed, reduced working memory capacity, impaired attention control, and diminished executive function, while vocabulary and semantic memory remain relatively preserved [137]. In animal models, aging is accompanied by spatial navigation deficits, motor impairment, and increased anxiety-like behavior [138]. These structural and behavioral changes reflect a progressive decline in the homeostatic capacity of brain networks and are accompanied by a series of deeply interconnected molecular and cellular hallmarks [137,138].

A growing body of evidence suggests that brain aging reflects a complex interplay between intrinsic genetic programs and extrinsic influences such as environmental exposures, leading to cumulative damage in both the neuronal and glial compartments. As reviewed by Lopez-Oudin (2023) [139], the major biological domains involved in this decline include genomic instability and progressive telomeric shortening, epigenetic changes, mitochondrial dysfunction and oxidative stress, disrupted Ca^2+^ signaling, impaired autophagy and protein homeostasis (proteostasis), neuroinflammation, vascular dysregulation, cellular senescence and reduced neurogenesis.

Together, these mechanisms impair intercellular communication and metabolic regulation, ultimately compromising the structural and functional resilience of neural tissue.

Age-associated mitochondrial alterations include the accumulation of somatic mitochondrial (mt)DNA mutations, decreased oxidative phosphorylation and increased reactive oxygen species (ROS) production, promoting redox imbalance, protein oxidation and activation of pro-inflammatory cascades [140,141]. These alterations compromise synaptic transmission and neuronal excitability, particularly in regions with high energy demand, such as the hippocampus and CBL. Impaired mitophagy, due to reduced activity of regulators such as PINK1, PARKIN, and NIX, further exacerbates mitochondrial dysfunction. Notably, experimental reactivation of mitophagy in aged mice and transgenic models of AD reduces protein aggregation and restores cognitive function [142].

Autophagy, a fundamental process responsible for the degradation and recycling of damaged proteins and organelles, declines markedly with age, contributing to altered proteostasis and chronic cellular stress. Neurons and glial cells exhibit reduced autophagosome biogenesis and lysosomal activity during aging, leading to progressive accumulation of misfolded proteins, dysfunctional organelles, and oxidative byproducts. For instance, defective autophagy is involved in the accumulation of neurotoxic proteins such as AMYLOID b, TAU, α-SYNUCLEIN, and TDP-43 [137,142]. Transcriptomic and proteomic studies report downregulation of key autophagy-related genes (e.g., *Atg7*, *Beclin-1*) and decreased lysosomal acidification in aged neurons and glia.

Importantly, astrocytes and microglia also exhibit reduced autophagic flux, which contributes to aberrant synaptic pruning, accumulation of lipid droplets, and chronic activation [142].

Ca^2+^ homeostasis is also disrupted during aging. Alterations include reduced buffering capacity, downregulation of RYRs and IP3 receptors, and impaired mitochondrial Ca^2+^ reuptake [137,143].

These changes alter synaptic plasticity, long-term potentiation (LTP), and neuronal network synchronization. Aged astrocytes display a reduced frequency and amplitude of spontaneous Ca^2+^ transients, further weakening the communication between glia and neuron [144].

Cellular senescence occurs in both neuronal and glial populations characterized by expression of markers such as p16^INK4A^ and senescence associated β galactosidase (SA-β-gal), secretion of senescence-associated secretory phenotype (SASP) factors, and DNA damage [37,143]. PCs in CBL and cortical neurons exhibit senescence-like features, including chromatin remodeling, telomere attrition, and nuclear envelope disruption. Senescent glia, especially astrocytes and microglia, promote low-grade neuroinflammation known as ‘inflammaging’. This condition contributes to synaptic loss, neuronal death, and impaired myelin regeneration [37,137]. Crosstalk between senescent glial cells, which involves cytokines, chemokines, and complement proteins, reinforces the organization of a pro-degenerative environment [3,41].

Vascular dysfunction contributes to aging-related decline. Structural changes in the NVU include mislocalization of astrocyte endfeets, reduced coverage of pericytes, and endothelial tight junction breakdown, resulting in BBB leakage [145]. These alterations allow infiltration of immune cells and alter neurovascular coupling, thus fueling metabolic dysregulation and neuroinflammation [146].

At the epigenetic level, single cell transcriptomic and epigenomic studies have shown widespread age-related changes in gene expression and chromatin organization. Glial cells show greater transcriptional responsiveness than neurons [37]. Hallmarks include DNA hypomethylation, altered histone modifications, and decreased chromatin accessibility, with functional consequences for glial reactivity, immune signaling, and energy metabolism [143].

Importantly, aging trajectories vary across brain regions. High-throughput single cell and spatial omics approaches reveal that aging signatures emerge earlier or more intensely in subcortical structures such as the CBL, hippocampus, and hypothalamus, compared to neocortical areas [5,37]. These observations highlight the region- and cell-type-specific nature of CNS aging, shaped by intrinsic developmental programs, local circuit properties, and extrinsic factors.

### 4.2. Glial Cell Aging

For decades, cognitive and motor decline associated with brain aging has been primarily attributed to progressive neuronal loss. However, quantitative and morphological studies have challenged this view, showing that neuronal numbers remain relatively stable in most brain regions during healthy aging [81,147,148]. Instead, increasing evidence points to functional deterioration of glial cells as a primary driver of brain vulnerability [137,143]. During aging, glial cells undergo profound cell type-specific and region-dependent changes that compromise their ability to maintain neural homeostasis. Recent transcriptomic studies reveal that glia, particularly in vulnerable regions such as the hippocampus, CBL, and hypothalamus, exhibit stronger and earlier transcriptional responses to aging compared to neurons [3,37]. These findings have led to a conceptual change: glia are no longer viewed as passive victims of aging, but as active contributors to age-related brain dysfunction. Understanding how glial aging shapes the CNS environment is therefore crucial for elucidating the mechanisms of brain aging and for the development of targeted interventions.

#### 4.2.1. Astrocytes

Astrocytes, historically considered passive support cells, are now recognized as active regulators of synaptic function, metabolic homeostasis and neuronal viability, CNS immunity, and brain resilience. During aging, they undergo substantial and regionally heterogeneous transformations that extend beyond morphological alterations to encompass transcriptional reprogramming and loss of homeostatic function. Although their overall number appears to be relatively preserved in most brain regions [147,148], astrocytes display marked molecular and functional changes [149] shaped by intrinsic aging processes and prolonged exposure to systemic and local stressors.

Bulk RNA-seq and snRNA-seq studies in rodents and humans have consistently identified astrocytes as the most transcriptionally responsive cell types during aging. Declining astrocytes downregulate genes involved in ion buffering (e.g., *Kcnj10*/*Kir4.1*), glutamate uptake (*Slc1a2*, *Slc1a3*), and cholesterol biosynthesis, while upregulating genes associated with immune activation, complement pathways (e.g., *C3*, *C4b*), and interferon signaling [3,5,149].

These changes indicate a shift toward a low-grade reactive state, distinct from the classical astrogliosis observed in acute injury.

Three-dimensional morphometric analyses of astrocytes in both human postmortem samples and rodent models have revealed age-associated structural remodeling. In the cortex and hippocampus, aged astrocytes exhibit reduced arbor complexity, retraction of fine processes, and decreased territorial coverage [150,151]. Astrocyte metabolism is also impaired by aging. Transcriptomic and lipidomic analyses of astrocytes from aged hippocampus and cortex reveal accumulation of lipid droplets, altered fatty acid metabolism, and impaired cholesterol biosynthesis, ultimately leading to reduced energetic support for neurons [3,152]. These effects are most pronounced in astrocytes located near WM or blood vessels, where metabolic demands are higher [149].

Furthermore, age-related mitochondrial dysfunction, characterized by reduced oxidative phosphorylation and increased mitochondrial fragmentation, further compromises astrocytic capacity to buffer redox stress and maintain neuron–glia metabolic coupling [153,154].

Alterations in Ca^2+^ signaling are another hallmark of astrocyte aging. In the hippocampus and CBL of aged rodents, astrocytes display a reduced frequency and amplitude of spontaneous Ca^2+^ transients, delayed responses to synaptic input, and dysregulated expression of Ca^2+^-handling proteins [144]. These deficits likely impair gliotransmission and compromise astrocytic modulation of synaptic activity.

In summary, astrocyte aging is a complex and multifactorial process characterized by transcriptional remodeling, reduced morphological complexity, metabolic dysfunction, and loss of neuroprotective functions. These changes do not occur uniformly, but are shaped by region, species, methodological variables, and environmental influences.

#### 4.2.2. WM and Oligodendroglia

One of the most evident alterations associated with CNS aging is WM atrophy, which is linked to myelin degeneration and reduced myelin renewal. These changes are correlated with motor and cognitive impairments [155,156,157]. Since OPCs are primarily responsible for myelin production and maintenance, numerous studies have investigated their age-related changes over time [158,159].

Several findings indicate that while OPCs remain numerically stable with age, they become functionally impaired and more heterogeneous [47,160]. Their mitotic activity decreases [161], and they exhibit a reduced ability to differentiate into OLs, reflecting the decreased ability of the CNS to regenerate myelin in old age. Older OPCs display slower differentiation rates and diminished responsiveness to pro-differentiation stimuli, partly due to decreased paracrine signaling and altered expression of critical receptors such as GPR17 and APJ [162,163].

In addition, quiescent adult OPC population undergo transcriptional remodeling with age, characterized by increased activation of immune and death cell pathway such as STAT1 and TGFβ signaling, and downregulation of cell cycle regulators such as MYC and KRAS [47].

OLs, which are highly long-lived cells, also exhibit age-related alterations [52,164]. Allen et al. (2023) [81] demonstrate through a single cell spatial transcriptomic study of the frontal cortex and striatum that the abundance of OLs identified by *Olig-2* expression decreased in old animals, while increasing the amount of those identified by *Olig-3* expression. These OLs are characterized by a transcriptional signature marked by upregulation of inflammatory and innate immune response genes such as *C4b* and *interleukin* 33 (*Il33*), and downregulation of myelin-associated transcripts, including *Mbp*, *Mog*, and *Mag* [81].

Conversely, a human study by Duffy et al. (2023) [165] which analyzes two cortical regions (entorhinal cortex and middle temporal gyrus) and two subcortical regions (putamen and subventricular zone) relevant for NDs, does not reveal a significant change in the proportions of oligodendroglia cells with age. However, the transcriptional alterations in aged human OLs are consistent with those observed in mice.

In addition, the functionality of oligodendroglia declines with aging, reflecting the decrease in myelin production, and the appearance of thinner and shorter internodes. The mechanisms underlying these changes are complex and involve many biological aspects.

Ols are particularly susceptible to oxidative stress due to their high metabolic activity, which leads to prolonged exposure to cytotoxic molecules such as ROS. These cells have also been shown to have reduced antioxidant levels compared to astrocytes and that these levels decline with age [166,167]. Mitochondrial dysfunction further exacerbates this vulnerability [168].

Oxidative damage affects all major macromolecules, including DNA, leading to mutations and genomic instability. As with neurons, both OLs and OPCs accumulate DNA damage with age due to inefficient DNA repair mechanisms [169]. Oxidative stress can also induce senescence in OPCs and OLs through multiple mechanisms, a process thoroughly reviewed by Spaas et al. (2021) [170].

This senescent phenotype is the result of intrinsic metabolic stress and the perturbed niche.

For instance, senescence is documented in other glial cells during aging [171], particularly microglia, which closely interact with OPCs and OLs in the maintenance of myelin.

Furthermore, Gomez et al. (2024) [172] demonstrate that the region-specific emergence of senescent OPCs in the WM of aged mice is not uniform throughout the brain. Specifically, they demonstrate the existence of regional phenotypes by comparing WM OPCs from the hippocampal fimbria with GM cells from the dental gyrus. Aged OPCs show increased cell size, reduced density, and closer proximity to microglia. These OPCs also exhibit a shared gene expression profile marked by upregulation of genes associated with senescence and OL disease (*Serp3n*, *Nol3*, *Skap2*), suggesting functional vulnerability and altered cell–cell interactions. This evidence provides useful targets for future investigations [172].

Epigenetic regulation of gene expression, which plays a crucial role in lineage specification during development [173], is also affected by aging in oligodendroglia. Human OLs exhibit significant hypomethylation of CpG islands with age, reflecting a global relaxation of epigenetic control [174].

Similarly, aged OPCs from the rat spinal cord display reduced expression of *Dnmt1* [175].

These alterations likely invalidate the transcriptional program necessary for remyelination. In aged mice, impaired recruitment of HDACs to inhibitory transcription factor promoters, such as *Sox2* and *Hes5*, results in increased expression of these factors, which affects the differentiation of OPCs [176].

These epigenetic modifications may be reversible and influenced by the niche environment.

Aging is associated with increased extracellular matrix stiffness [177], and the accumulation of inhibitory molecules, such as hyaluronan and chondroitin sulfate proteoglycans, which impair the proliferation and differentiation of OPCs [178].

Cell–cell interactions are also recognized as important regulators of myelin health.

Microglia, astrocytes, and OLs could be considered a functional unit [69], with astrocytes and OLs physically interacting through connexins [179]. With age, both astrocytes and microglia adopt a senescence-associated secretory phenotype, as illustrated before, which creates a pro-inflammatory microenvironment detrimental to OL viability and function.

Recent studies (reviewed in [155]) have also revealed an increase in the infiltration of adaptive immune cells, particularly cytotoxic CD8^+^ T cells, in aged WM, especially in areas close to the lateral ventricles [180]. These cells are often located near OLs and microglia and trigger an IFNg-responsive state in both cells, ultimately leading to myelin loss. These findings underscore the complexity of cell–cell interactions in aging and their contribution to impaired OPC differentiation and OL function.

#### 4.2.3. Microglia

Recent studies have revealed that microglia are highly dynamic and plastic cells that can undergo transitions along a continuum of functional states in response to intrinsic and extrinsic cues.

Aging has emerged as a key factor in modulating this plasticity [78,83,181]. Transcriptomic, proteomic, and morphological analyses have been instrumental in identifying aging-associated features of microglia. Given the involvement of these cells in the pathogenesis of several NDs, multiple transcriptomic studies have been performed in both mouse models and postmortem human tissue. Olah et al. (2018) [182] provided the first transcriptomic atlas of an older human dorsolateral prefrontal cortex, showing that microglia exhibit a distinct gene expression profile enriched for pathways related to DNA damage, telomere maintenance, and phagocytosis.

Importantly, many of these genes overlap with those found in Alzheimer disease (AD) and MS-associated profiles [182]. Interestingly, these transcriptomic changes are accompanied by parallel proteomic changes. Subsequent studies confirmed that aged microglia exhibit a pro-inflammatory transcriptional signature along with downregulation of homeostatic genes, including those involved in phagocytosis. These changes impair the clearance of apoptotic cells and myelin debris, a hallmark of aging [183,184,185].

Age-associated alterations in myelin contribute to the emergence of distinct microglia phenotypes in WM, known as WM-associated microglia (WAM). This state shares molecular features with disease-associated microglia (DAM) [186]. WAM are typically found in clusters or nodules engulfed with aberrant myelin and may initially exert adaptive and protective functions.

Successively, prolonged microglia activation promotes CD8^+^ T cell recruitment, triggering axonal degeneration and myelin loss [155] (see also Section 3.2). A hallmark of aged microglia is the acquisition of dystrophic morphology characterized by reduced ramification, cytoplasmic swelling, and fragmentation [187]. Although this morphology is primarily associated with pathology rather than aging per se [188], it frequently overlaps with the molecular features of senescence.

These features include increased p16^INK4A^ expression, lipofuscin accumulation, SA-β-Gal activity and SASP components. Sex-specific microglial aging trajectories have also been described (reviewed in [189]). Sex differences in the microglia transcriptome have been linked to differential regulation of the immune process, cytokine-mediated signaling pathway, and phagocytosis. Female cells undergo a progressive aging process, whereas male microglia show a more abrupt transition [190]. The underlying mechanisms are not well understood, but they may contribute to sex-biased vulnerability to NDs. Finally, there is an ongoing debate about the need for a unified system for classifying and interpreting microglial states in different brain regions and conditions [17,83,181,191].

Table 1 schematizes the most relevant evidence synthesizing the altered functions of glial cells in the CNS aging.

## 5. Cerebellar Aging

### 5.1. Hallmarks of Cerebellar Aging

With aging, the CBL undergoes different anatomical, molecular, and functional changes that affect balance, coordination, cognitive flexibility, and associative learning [192,193,194]. Although the overall loss of cerebellar volume appears to be less pronounced than that observed in the cortex, increasing evidence indicates that aging affects the CBL in a region-specific and cell-type-specific manner.

Early postmortem stereological analysis shows that the human CBL exhibits selective structural changes, with pronounced degeneration in the anterior lobe, a region involved primarily in motor control [195]. These changes included a ~40% reduction in GCs and PCs, a loss of cortical and WM volume, and a reduction in PC soma size in the absence of nuclear loss.

Wang et al. (2024) [196], integrating volumetric and morphological analyses and applying a specific algorithm to divide the organ into different subregions, obtain a more complete picture of cerebellar aging. The authors confirm a progressive reduction in cerebellar volume with age, including deep WM. Atrophy was most pronounced in vermis X, the lateral regions of the cerebellar hemispheres, the I-III lobules, and the medial portions of the posterior lobe.

Notably, although men generally have larger volumes than women in most regions of the CBL, they show slightly more pronounced age-related atrophy, particularly in the right crus II [196].

Cross-sectional data limit our understanding of temporal dynamics. However, few longitudinal studies of healthy individuals have also reported cerebellar shrinkage and region-specific atrophy [197,198]. Complementary approaches highlight the associations between preserved cerebellar structure and cognitive performance. For instance, Feng et al. (2025) [199] discovered that, among all regions of the brain examined, the VIIb lobule of the right CBL showed the strongest correlation with preserved common executive functions in aging adults.

Age-related functional reorganization has also been observed. Functional connectivity MRI (fcMRI) studies demonstrate that reduced volumes in Crus I/II and Vermis VI are associated with impaired working memory, attention, and executive function [200]. Task-based fcMRI reveals that older adults exhibit more diffuse and less distinct cerebellar activation patterns.

Cognitive tasks recruit motor-related regions (e.g., lobule V) and motor tasks engage cognitive regions (e.g., Crus I/II), indicating a form of compensatory scaffolding [201,202] that is similar to that observed in the prefrontal cortex [193]. Furthermore, aging results in less lateralized and more symmetric cerebellar activation, reflecting reduced topographic precision and efficiency of cerebellar-cortical integration [203]. Despite these indications of vulnerability, the CBL maintains a notable degree of structural and functional resilience and appears to resist the development of specific alterations in the early stage of NDs such as AD [201,204]. Very recent proteomic profiling of AD tissues indicates that the CBL displays higher expression levels of synaptotropic genes compared to the cerebral regions. This elevated expression is believed to confer neuroprotection, which can protect the CBL during dementia or AD progression [205].

All structural and functional alterations observed in CBL aging are accompanied by specific cellular changes.

PCs, which have high metabolic demands, are particularly susceptible to the effects of aging. They exhibit dendritic atrophy, spine loss, and reduced electrophysiological precision [206,207]. Woodruff-Pak et al. (2010) [207] observe selective PC loss, particularly in the vermis. Donofrio et al. (2025) [118] recently demonstrated that PC loss in aged mice occurs in a parasagittal pattern. They also found evidence of regionally selective PC loss in human postmortem samples.

Cerebellar aging involves not only neuronal atrophy but also changes in the cellular phenotype of neural tissue that could precede death.

Mitochondrial dysfunction is a recurring hallmark in the cerebellar compartments. Electron microscopy in rodents reveals that mitochondrial alterations occur early and selectively in specific types of cerebellar cells. In PCs, aging is associated with a reduced number of mitochondria, [208]. Functional impairments are also evident. The activity of cytochrome c oxidase (complex IV) declines significantly in an older CBL, [209]. Increased oxidative stress and reduced respiratory efficiency have also been described in neurons and astrocytes [210].

Ca^2+^ signaling is another critical domain affected by aging. PCs rely on tightly regulated intracellular Ca^2+^ signaling to maintain excitability, synaptic integration, and motor coordination.

In parallel, BG in aged mice exhibits increased spontaneous Ca^2+^ waves, which can perturb PC firing, particularly in lobule VI and the anterior vermis [211]. Notably, BG modulates PC excitability through Ca^2+^-dependent K^+^ uptake, which contributes to regulation of PC bistability and firing dynamics, highlighting the importance of glial Ca^2+^ signaling in shaping cerebellar output [212].

Autophagy dysfunction contributes to the accumulation of intracellular debris. Furthermore, autophagy dysfunction is emerging as a hallmark of age-related cerebellar diseases, such as Spinocerebellar Ataxias (SCAs) and mitochondrial disorders, where defective autophagy exacerbates PC degeneration [213,214]. Aging-related myelin atrophy is evident in human cerebellar WM, particularly within the cerebellar peduncles and DCN [215]. These changes are a consequence of the alterations that oligodendroglia undergo. They will be discussed in the following.

Although neurovascular and glymphatic dysfunctions are increasingly recognized as contributing factors to brain aging, specific data on the CBL remain limited. Only a few rodent studies have characterized cerebellar aging at the glial-vascular interface. Aged mice exhibit significant upregulation of KIR4.1 and AQP4 in cerebellar astrocytes [216] as well as a notable increase in spontaneous Ca^2+^ waves in BG, accompanied by local tissue hypoxia [211]. Together, these findings support the hypothesis that cerebellar NVU may become progressively dysregulated with age, potentially altering metabolic homeostasis and glial-vascular signaling.

### 5.2. Glial Cells Aging

Research on cerebellar aging has historically focused on neurons, particularly PCs. However, accumulating evidence now suggests that neuronal dysfunction in the aging CBL reflects broader changes in the cellular microenvironment. Specifically, signs of oxidative stress, mitochondrial dysfunction, and impaired homeostasis have been observed not only in neurons but also in cerebellar glial populations [205,207,217]. In some cases, these glial changes appear to precede or exacerbate neuronal vulnerability, raising the hypothesis that glial senescence may be a primary driver of cerebellar aging. Given their critical roles in synaptic regulation, immune surveillance, metabolic support, and myelin maintenance, cerebellar glial cells may represent a focal point in aging research.

The following sections explore how specific glial subtypes undergo structural and functional changes with age and how these alterations may contribute to cerebellar decline.

#### 5.2.1. Astrocytes

Astrocytic aging in the CBL is increasingly recognized as a contributing factor to cellular and network-level dysfunction. A growing body of evidence indicates that aged astrocytes undergo a progressive remodeling of gene expression, morphology, and signaling capacity. Among them, BG exhibits altered Ca^2+^ dynamics, metabolic fragility, and early signs of reactive transformation.

One of the most comprehensive data sets is provided by Boisvert et al. (2018) [3], who analyzed astrocytes from multiple regions of the brain, including the CBL, in young and old mice using RiboTag RNA-seq. Transcriptomic profiling reveals that cerebellar astrocytes exhibit the most extensive changes in age-related gene expression between all regions analyzed, with more than 400 differentially expressed genes. These include upregulation of reactivity-associated markers, such as *Gfap*, *Serp3n*, as well as immune-related transcripts, such as *C3*, *C4b*, consistent with a shift towards a primed, moderate inflammatory state. They do not show upregulation of key interferon-responsive cytokines (e.g., *Cxcl10* and *Il6*), nor do they significantly induce phagocytic receptors such as *Mertk* or *Megf10* [3]. Instead, the older CBL appears to adopt a ‘permissive’ environment for synaptic remodeling, inducing in astrocytes the increased expression of *Sparc*, *Thbs2*, and *Tgfβ2*, without acquisition of a neurotoxic state similar to A1 [3]. More recent transcriptomic studies support these findings, reporting evidence of neuroimmune priming in aging astrocytes, including upregulation of MHC-I components (e.g., *H2-K1*, *Tap1*) and interferon-stimulated genes [13,218].

While this profile does not fully align with the canonical A1 signature [41], it shares several pro-inflammatory features and may represent a partially reactive CBL-specific state. In addition, Tsai et al. (2025) [13] also report a reduction in spatial proximity between aged microglia and astrocyte subtypes, suggesting altered glial communication. Although BG morphology was not directly assessed in that study, the findings support a model of multicellular remodeling in the older CBL. Despite substantial transcriptional changes, the core astrocytic homeostatic genes, including *Slc1a2* (*Glt1*), *Aqp4*, *Gja1* (*connexin-43*), *Glul*, and *Kcnj10* (*Kir4.1*), remain largely stable, indicating preserved capacity for synaptic support and metabolic regulation. In contrast, genes involved in cholesterol biosynthesis pathways are significantly downregulated (e.g., *Hmgcr*, *Fdps*, *Sqle*), whereas cholesterol transport genes (e.g., *ApoE* and *Abcg1*) are upregulated, suggesting an adaptive remodeling of membrane metabolism in the aging CBL.

Taken together, these findings indicate that although aging induces significant transcriptomic remodeling in cerebellar astrocytes, many core functions are preserved. These age-related traits should be interpreted considering the intrinsic astrocyte identity of the CBL, which differs from that of other regions of the brain in adulthood. Even in adult mice, cerebellar astrocytes differ substantially from cortical populations, exhibiting higher basal expression of genes involved in synaptic regulation (e.g., *Thbs1-4*, *Sparcl1*, and *C3*) and structural components (e.g., *Gfap*, *Aqp4*, *Vim*), while maintaining stable levels of key transporters (*Slc1a2*, *Slc1a3*, and *Slc6a11*) and metabolic enzymes (*Glul*). This distinct molecular signature likely contributes to their differential vulnerability and response to aging [3]. These transcriptomic findings are supported by morphological and physiological data from rodent models. In aged rats, cerebellar astrocytes show features of cellular senescence, including hypertrophy and hyperplasia, increased GFAP immunoreactivity and lipofuscin accumulation, hallmarks of astrocytic senescence [219].

Although some studies report modest increases in GFAP in cortical and subcortical regions with aging [220], similar changes in the CBL have yet to be consistently demonstrated. Nevertheless, the absence of overt astrocyte loss or gliopathy suggests the presence of region-specific, low-grade astrogliosis in the aging CBL. In this context, BG may play a central role in preserving cerebellar architecture. Although the aging trajectory of BG remains poorly defined, recent scRNAseq analyses reveal that BG are enriched in genes involved in synaptic organization, glutamate homeostasis, and metabolic coupling with neurons [27]. This unique transcriptional profile suggests that any age-related perturbation, such as oxidative stress, energy deprivation, or neurotransmitter imbalance, is more likely to impact BG, potentially triggering early astroglial dysfunction in the cerebellar cortex. This hypothesis is consistent with the astroglia alterations observed in ML of a murine model of aging induced by chronic exposure to D-galactose that causes oxidative stress along with significant PC degeneration and cortical thinning.

Due to their highly specialized morphology, radial organization and proximity to PCs, BGs are likely involved in early remodeling during aging. Spatial transcriptomic data show that BG form a distinct transcriptional cluster enriched in lobules VI, IX, and X of the mouse CBL, reinforcing this hypothesis [87]. These lobules are characterized by high metabolic demand and functional complexity. In particular, the same posterior regions show early signs of morphological remodeling and functional decline in the CBL aging of both humans and rodents and are involved in sensorimotor and cognitive integration [194]. This regional enrichment suggests that BG may be differentially susceptible to age-related metabolic, synaptic, or vascular stressors, even in the absence of obvious morphological degeneration.

Experimental models provide functional insights into BG behavior under stress. In acute metabolic stress, such as oxygen and glucose deprivation, these cells respond rapidly with membrane depolarization and Ca^2+^ influx, but exhibit limited recovery, indicating metabolic fragility [221]. Chronic alterations in BG Ca^2+^ dynamics, such as increased spontaneous transients and prolonged responses, have been linked to impaired PC bistability and reduced firing precision, particularly in motor-related lobules such as lobule VI and the anterior vermis [211,222]. Several lines of evidence suggest that aging may alter the fine-tuned interaction between BG and PCs. In aged mice, BG exhibits up to 20-fold increase in spontaneous Ca^2+^ transients under hypoxic conditions, which is interpreted as a sign of impaired metabolic and signaling homeostasis.

In SCAs, BG exhibit early dysfunction, including reduced expression of KIR4.1, GLAST, and adhesion molecules, leading to impaired glutamate clearance and excitotoxic stress in PCs [223,224]. Notably, glial reactivity and glia-synaptic disruption often precede neuronal degeneration, supporting the idea that astrocytic pathology makes a primary, cell-autonomous contribution to cerebellar disorders and aging.

#### 5.2.2. WM and Oligodendroglia

Cerebellar WM undergoes structural alteration during aging. However, data on the extent and distribution of these changes are inconsistent due to differences in experimental design and age ranges in the studies [225,226,227]. WM loss related to aging is frequently accompanied by the emergence of focal lesions that appear hyperintense on MRI (WM hyperintensity, WMH). In humans, DTI studies have documented age-related reductions in the integrity of cerebellar WM, especially in the fronto-cerebellar tracts [134]. Both the number and the volume of WMHs have been correlated with changes in brain connectivity, including in cerebellar networks [228,229].

Despite the growing interest in the involvement of the cerebellar WM in NDs and in late-onset SCA, notably type 6, which, unlike most hereditary ataxias, is largely restricted to the CBL [230], the response of oligodendroglial cells during healthy aging remains poorly understood.

While the role of adaptive immune reactions in myelin pathology has been explored in other regions of the brain, the CBL has received limited attention in this context. Nonetheless, it is known to be susceptible to autoimmune disorders [231], and one proposed mechanism involves the distinctive features of the blood–cerebrospinal fluid barrier (BCSFB), which may allow immune cell infiltration via the choroid plexus of the fourth ventricle and the pial vasculature between folia.

#### 5.2.3. Microglia

Age-related microglia dystrophy is particularly evident in the CBL, as shown in murine models, where these cells exhibit process thickening, deramification of processes, and hypertrophy of the cell body. Such changes are especially evident in the WM of the inferior cerebellar peduncles, where microglia aggregate and show markedly thickened processes [232]. Beyond morphological alterations, cerebellar microglia also undergo substantial transcriptional remodeling with aging among different cell types.

Hahn et al. (2023) [12] identified a common aging signature (CAS) in mouse brain RNA, comprising 82 genes primarily related to immune and inflammatory pathways, many of which are involved in microglial regulation. The CBL showed one of the highest rates of changes in CAS expression, comparable to the striatum and caudate putamen and more pronounced than those of the cortex. Notably, CAS-related gene expression increased in aged brain regions, reaching a greater extent in cerebellar microglia [12].

These findings are consistent with previous data from Grabert et al. (2016) [11], who reported that adult cerebellar microglia exhibit increased immunological responsiveness and sensitivity to aging compared to those of other brain regions. This is characterized by elevated expression of interferon-responsive genes and cytokine-mediated signaling pathways.

Very recently, Tsai et al. (2025) [13], analyzing sorted cerebellar microglia isolated from aged mice, obtain an RNA signature coincident with a neuroprotective functional state [13] corresponding to upregulation of *ApoE*, *Stat1*, *Axl*, alongside the downregulation of genes involved in lipid droplet accumulation, features characteristic of AD-related degeneration [233].

Interestingly, applying multiplexed error-robust fluorescence in situ hybridization (MERFISH), to localize genes relevant for aging, neuroinflammation and cellular stress, Tsai et al. (2025) [13] further reveal a distinct, age-related, spatial redistribution of microglia in the CBL. In aged mice, microglia are predominantly localized within GL, in proximity to GCs, and expressed genes such as *Cbln1*, *Cdk2*, and *Cxcl2*, suggesting a region-specific adaptive response to aging. The molecular phenotype of aged microglia may initially suggest a protective state that supports debris clearance and homeostatic maintenance. However, with time and sustained stress, this phenotype may shift toward a more reactive and damage-prone state. Notably, many of these ‘activated’ molecular traits are also observed in aging cerebellar astrocytes [3,4,32], raising the hypothesis of a coordinated multicellular neuroinflammatory response in the older CBL.

The CBL is increasingly recognized as a critical node in NDs [234]. In the early stages of NDs, microglia proliferate and exert neuroprotective functions. However, as the disease progresses, microglia adopt a pro-inflammatory phenotype [235,236,237,238], leading to increased neuronal vulnerability and degeneration. Several evidence show an increased number of microglia cells in the CBL of AD patients [239,240,241], as well as in relevant animal models and this cells express *Trem2*, a gene involved in the resiliency to AD pathogenesis in CNS [242]. Nonetheless, although the CBL appears to be more resilient to neurodegeneration than other regions of the brain, the precise mechanisms underlying this resistance need to be fully elucidated.

Table 2 summarizes the most significant findings regarding the altered functions of glial cells in CBL aging.

## 6. Conclusions

This review highlights region-specific vulnerabilities and unexpected resilience capacities during CBL aging. While brain aging is determined by the cumulative impact of environmental, biological, and behavioral exposures, collectively called the ‘exposome’, the CBL follows a distinct trajectory, marked by selective glial remodeling and relative structural preservation.

**Figure 1:**
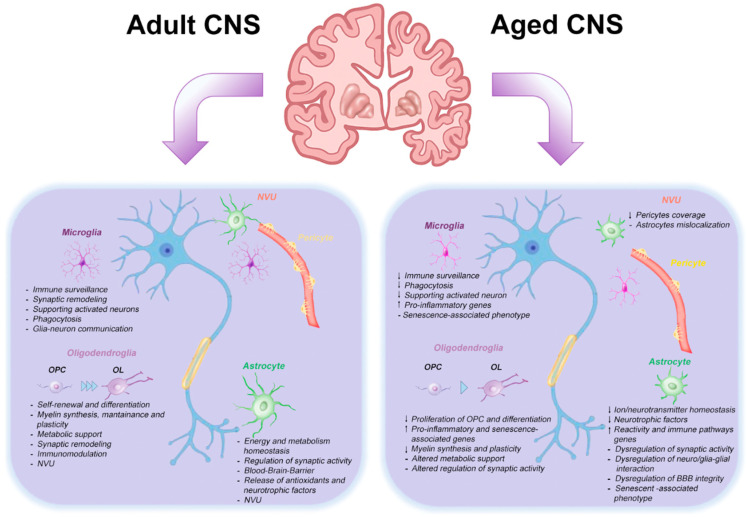
Hallmarks of CNS aging. The glial cells’ characteristics in adult and healthy older CNSs are schematized. NVU: neurovascular unit; OPC: oligodendrocyte progenitor cell; OL: mature oligodendrocyte.

As illustrated in Figure 1, which schematizes the main glial alterations in the adult and older CNS, aging affects glial cell populations in a coordinated yet regionally diversified manner. These general changes are particularly relevant when comparing the cerebellum to other CNS regions.

The highly laminar architecture, dense synaptic organization, and functional compartmentalization across the lobules provide a uniquely defined anatomical context in which interactions between glia and neurons can be examined with spatial precision. These features make the CBL a valuable system for investigating how glial cells mediate protective versus maladaptive responses across subregions involved in motor and cognitive functions.

**Figure 2:**
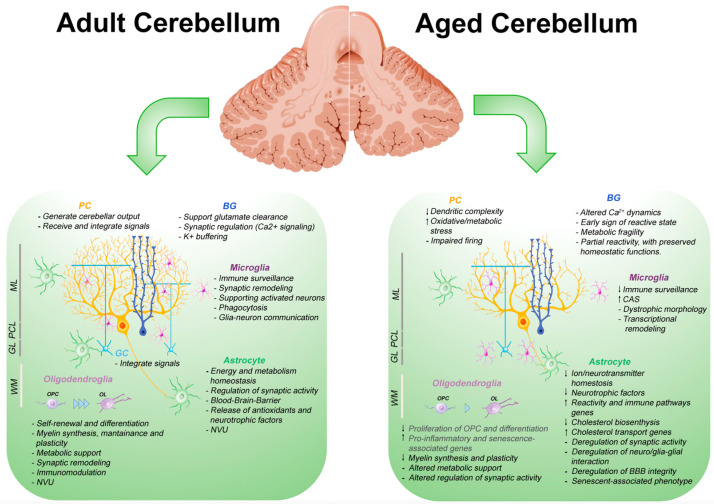
Hallmarks of the CBL aging. The glial cells characteristics in adult and healthy aged CBL are schematized. Cortical layers of the mature CBL are labeled on the right as follows: ML: molecular layer; PCL: Purkinje cell layer; GL: granule layer; WM: white matter. PC: Purkinje cell; GC: granule cell; BG: Bergman Glia; OPC: oligodendrocyte progenitor cell; OL: mature oligodendrocyte; NVU: Neurovascular unit; CAS: common aging signature. Statements in gray are speculative and derived from pathological models or extrapolated from cortical data, due to limited evidence in the cerebellum.

Comparative analyses reveal significant differences in glial aging between the CBL and other regions of the CNS. As summarized in Figure 2, cerebellar astrocytes, especially BG, display a distinct transcriptional profile characterized by moderate reactivity and partial immune priming without transitioning to a fully neurotoxic state. BG undergo early and localized remodeling, particularly in Ca^2+^ dynamics and synaptic regulation, while maintaining key homeostatic functions, suggesting controlled adaptation rather than degeneration.

Furthermore, microglial aging in the CBL is distinct. These cells exhibit an accelerated expression of CAS and undergo spatial redistribution, especially in GL. This may reflect enhanced neuroimmune surveillance or an adaptive effort to preserve tissue integrity.

Oligodendroglial changes are less well defined, but initial findings suggest region-specific vulnerabilities in WM integrity, with potential implications for NDs.

Despite these cellular changes, the CBL maintains substantial anatomical and functional integrity throughout its lifetime. Neuroimaging studies demonstrated that there is a reduction in circuit segregation and topographic specificity with age, along with increased recruitment of compensatory networks, supporting the notion of a cerebellar cognitive reserve.

However, there are still important questions to answer. The boundary between adaptive glial remodeling and functional decline remains unclear. The reversibility of these glial states and their potential as therapeutic targets are open questions, as are the roles of sex differences and lobular specificity in shaping aging trajectories. To address these issues, future research should integrate spatially resolved, multi-omics approaches with advanced human-relevant models, including *human induced pluripotent stem cells* (hiPSC)-derived cerebellar glial cells, cerebellar organoids, long-term glia-neurons co-cultures, and chimeric systems. These tools will be essential to clarify how glial cells contribute to cerebellar aging. Finally, recent evidence indicates that the CBL displays increased baseline expression of synaptotropic and neuroprotective genes and exhibits relative preservation in early AD, suggesting the importance of studying the role of glial networks in supporting resilience. Identification of CBL-specific biomarkers of glial aging has the potential to develop new strategies for the preservation of brain function in aging populations.

A concise summary box of the main concepts discussed in this review is provided below.

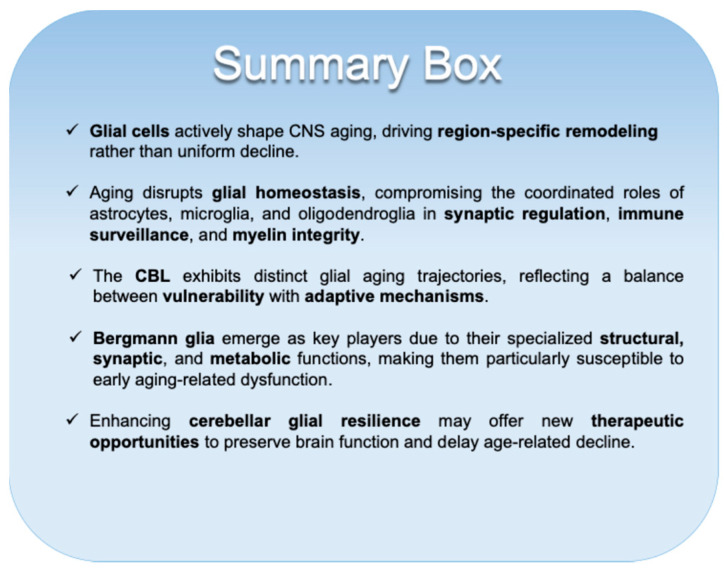



## Figures and Tables

**Table 1:** Main Functions and aging-related changes in CNS glial cell types.

Cell Type	Adult CNS Main Functions	Aged CNS Main Changes
**Astrocytes**	- Energy and metabolic homeostasis (lactate, pyruvate, ion regulation) - Synaptic regulation (Ca^2+^-dependent signaling) - BBB support - Release antioxidants and neurotrophic factors	- Impaired neuronal metabolic support (lipid droplets accumulation, altered fatty acid/cholesterol metabolism) - Altered Ca^2+^ signaling - Impairment of BBB integrity -Upregulation of GFAP (astroglial reactivity)
**Microglia**	- Immune surveillance - Apoptotic cell clearance (phagocytosis) - Neuroprotection - Synaptic remodeling/pruning	- Upregulation of immune/inflammatory genes - Downregulation of homeostatic/phagocytic genes - Morphological dystrophy (reduced ramifications, cytoplasmic swelling, fragmentation) - Senescence-associated phenotype (increased p16^INK4A^ expression, lipofuscin accumulation, SA-b-Gal activity and SASP components)
**OPCs**	- Self-renewal (progenitors pool) - Myelin maintenance - Differentiation into OLs for remyelination	- Reduced mitotic activity and differentiation into OLs - Increased susceptibility to senescence signals
**Oligodendrocytes (OLs)**	- Myelin synthesis and plasticity - Axonal metabolic support (e.g., K^+^ homeostasis) - Maintenance of WM integrity	- Decline in myelin regeneration - Altered metabolic support - Altered synaptic activity - Upregulation of immune/inflammatory pathways

**Table 2:** Main functions and aging-related changes in CBL glial cell types.

Cell Type	Adult CBL Main Functions	Aged CBL Main Changes
**Bergmann Glia**	- Structural support and maintenance of Purkinje cell (PC) dendrites - Synaptic regulation (Ca^2+^ dynamics, AMPA/GABA-A receptor response, enwrapment of PC synapses) - Neurotransmitter clearance (glutamate uptake through GLAST, GLT-1; glutamine production) - Ion and metabolic homeostasis (K^+^ buffering via KIR4.1; water regulation via AQP4; metabolic coupling with PCs)	- Partial reactive phenotype: upregulation of GFAP, SERPIN3n, C3, C4b; mild neuroimmune activation without full A1 profile - Altered Ca^2+^ dynamics and metabolic fragility: increased spontaneous Ca^2+^ transients - Disrupted synaptic and glia-neuron interactions: reduced KIR4.1 and GLAST, impaired glutamate clearance, disturbed PC firing precision
**Astrocytes (Velate, Fibrous, Vascular)**	- Energy and metabolism homeostasis (e.g., glutamine production, metabolic coupling with PCs) - Regulation of synaptic structure and plasticity - Neurovascular support and BBB integrity - Release of antioxidants and neurotrophic factors	- Reduced cholesterol biosynthesis - Dysregulated glia–neuron interaction - Impairment of BBB integrity and neurovascular uncoupling (astrocyte mislocalization) - Increased expression of immune- and inflammatory-related gene (Gfap, *C*3, *Mhc*-I)
**Microglia**	- Immune surveillance and phagocytosis (apoptotic cell clearance, especially PCs) - Region-specific transcriptional profile with enriched expression of genes involved in immune alertness (e.g., pathogen recognition, antigen presentation) - Regulation of synaptic refinement (pruning)	- Morphological dystrophy: hypertrophy, process thickening, ramification loss (especially in WM) - Increased CAS genes expression (e.g., those involved in immune/inflammatory pathways) - Increased interferon-responsive and cytokine-mediated signaling - Higher sensitivity to stress and disease-associated degeneration
**OPCs/Oligodendrocytes**	- Myelination of PC axons and cerebellar WM - Support of synaptic transmission and circuit precision - Axonal metabolic support (lactate/pyruvate release for energy supply in synergy with astrocytes)	- Structural alterations of cerebellar WM - Loss of myelin integrity and synaptic synchronicity - Poorly characterized oligodendroglial response in aging (limited data compared to cortex) - Potential vulnerability to immune infiltration

## Abbreviations

CBL: Cerebellum; CNS: Central Nervous System; WM: White matter; GM: gray matter; ND: Neurodegenerative Disease; BBB: Blood–brain barrier; RGC: Radial glia cell; OPC: Oligodendrocyte Precursor Cell; OL: Oligodendrocyte; BAM: Border-Associated Macrophage; CSF: Cerebrospinal Fluid; CC: Corpus Callosum; GO: Gene Ontology; TRAP: Translating Ribosome Affinity Purification; scRNA-seq: single cell RNA-sequencing; snRNA-seq: single nuclear RNA-sequencing; FA: Fibrous Astrocyte; PAP: Perisynaptic Astrocytic Process; NVU: Neurovascular Unit; ML: Molecular Layer; PCL: Purkinje Cell Layer; PC: Purkinje Cell; GL: Granular Layer; GC Granule Cell; DCN: Deep Cerebellar Nuclei; CF: Climbing Fiber; IO: Inferior Olive; PF: Parallel Fiber; MF: Mossy Fiber; BG: Bergmann Glia; VA: Velate astrocyte; VZ: Ventricular Zone; SHH: Sonic Hedgehog; AP: action potential; MS: Multiple Sclerosis; MRI: Magnetic Resonance Imaging; DTI: Diffusion Tensor Imaging; rs-fMRI: resting-state functional MRI; fcMRI: functional connectivity MRI; mt: mitochondrial; ROS: Reactive Oxygen Species; LTP: Long-Term Potentiation; SASP: Senescence-Associated Secretory Phenotype; AD: Alzheimer Disease; WAM: WM-associated microglia; DAM: Disease-Associated Microglia; SCA: SpinoCerebellar Ataxia; WMH: WM hyperintensity; BCSFB: Blood–Cerebrospinal Fluid Barrier; CAS: Common Aging Signature; MERFISH: Multiplexed Error-Robust Fluorescence In Situ Hybridization; hiPSC: human induced Pluripotent Stem Cell.

## List of Full Gene and Protein Names

**Genes**	
*Abcg1*	ATP Binding Cassette Subfamily G Member 1
*Aldoc* (*Zebrin II*)	Aldolase C
*Atg7*	Autophagy Related 7
*Beclin-1*	Beclin 1
*C3*	Complement Component 3
*C4b*	Complement Component 4B
*Cbln1*	Precerebellin 1 Precursor
*Cdk2*	Cyclin Dependent Kinase 2
*C-fos*	FBJ Murine Osteosarcoma Viral Oncogene
*C-jun*	JUN Proto-Oncogene
*Cxcl10*	C-X-C Motif Chemokine Ligand 10
*Cxcl2*	C-X-C Motif Chemokine Ligand 2
*Fdps*	Farnesyl Diphosphate Synthase
*Gfap*	Glial Fibrillary Acidic Protein
*Glul*	Glutamate-Ammonia Ligase
*H2-K1*	Histocompatibility 2, K1, K Region
*Hes5*	Hes family bHLH transcription factor 5
*Hmgcr*	3-Hydroxy-3-Methylglutaryl-CoA Reductase
*Il33*	Interleukin 33
*Il6*	Interleukin 6
*Kcnj10*	Potassium Inwardly Rectifying Channel Subfamily J Member 10
*KRAS*	Kirsten Rat Sarcoma Viral Oncogene Homolog
*Lama2*	Laminin Subunit Alpha 2
*Megf10*	Multiple EGF Like Domains 10
*Mertk*	MER Proto-Oncogene, Tyrosine Kinase
*Mbp*	Myelin Basic Protein
*Mag*	Myelin Associated Glycoprotein
*Mog*	Myelin Oligodendrocyte Glycoprotein
*Mybpc1*	Myosin Binding Protein C1
*Nol3*	Nucleolar Protein 3
*Olig1*	Oligodendrocyte Transcription Factor 1
*Olig-2*	Oligodendrocyte Transcription Factor 2
*Olig-3*	Oligodendrocyte Transcription Factor 3
*Pdgfr-a*	Platelet Derived Growth Factor Receptor Alpha
*Sept4*	Septin 4
*Serp3n*	Serpin Family 3 Member N
*SHH*	Sonic Hedgehog
*Skap2*	Src Kinase Associated Phosphoprotein 2
*Slc1a2*	Solute Carrier Family 1 Member 2 (EAAT2)
*Slc1a3*	Solute Carrier Family 1 Member 3 (EAAT1)
*Slc6a11*	Solute Carrier Family 6 Member 11
*Sox2*	SRY-Box Transcription Factor 2
*Sparc*	Secreted Protein Acidic and Rich in Cysteine
*Sqle*	Squalene Epoxidase
*Tgfβ2*	Transforming Growth Factor Beta 2
*Thbs1–4*	Thrombospondin 1 to 4
*Thbs2*	Thrombospondin 2
*Vim*	Vimentin
*Wif1*	WNT Inhibitory Factor 1
*Zebrin II*	see *Aldoc*
**Proteins**	
**AMPAR**	α-Amino-3-Hydroxy-5-Methyl-4-Isoxazolepropionic Acid Receptor
**APJ**	Apelin Receptor
**ApoE**	Apolipoprotein E
**AQP4**	Aquaporin 4
**C3**	Complement Component 3
**C4b**	Complement Component 4B
**CALB**	Calbindin
**GABA**	Gamma-Aminobutyric Acid
**GLAST**	Glutamate Aspartate Transporter
**GLT-1**	Glutamate Transporter 1
**GJA1**	Gap Junction Alpha-1 Protein (Connexin-43)
**GPR17**	G Protein-Coupled Receptor 17
**HDACs**	Histone Deacetylases
**IP3**	Inositol 1,4,5-trisphosphate
**KIR4.1**	Inward Rectifier Potassium Channel 4.1
**MAG**	Myelin-Associated Glycoprotein
**MBP**	Myelin Basic Protein
**MOG**	Myelin Oligodendrocyte Glycoprotein
**MYC**	MYC Proto-Oncogene
**NG2**	Neural/Glial Antigen 2
**NIX**	BNIP3-Like Protein
**NOTCH**	Notch Receptor
**P16^INK4A^**	Cyclin Dependent Kinase Inhibitor 2A
**PARKIN**	E3 Ubiquitin-Protein Ligase Parkin
**PDGFRα**	Platelet Derived Growth Factor Receptor Alpha
**PINK1**	PTEN-Induced Kinase 1
**PTCH1/2**	Patched 1 and 2 Receptors
**RYR3**	Ryanodine Receptor 3
**SA-β-Gal**	Senescence-Associated β-Galactosidase
**SHH**	Sonic Hedgehog
**STAT1**	Signal Transducer and Activator of Transcription 1
**TAP1**	Transporter Associated with Antigen Processing 1
**TDP-43**	TAR DNA Binding Protein 43
**TAU**	Microtubule-Associated Protein Tau
**TGFβ**	Transforming Growth Factor Beta
